# Investigation into the Role of Long-Non-Coding RNA *MIAT* in Leukemia

**DOI:** 10.3390/ncrna9040047

**Published:** 2023-08-11

**Authors:** Alessia Ostini, Mirna Mourtada-Maarabouni

**Affiliations:** School of Life Sciences, Faculty of Natural Sciences, Keele University, Newcastle-under-Lyme ST5 5BG, UK; a.ostini@keele.ac.uk

**Keywords:** non-coding RNA, lncRNA, Myocardial Infarction Associated Transcript (*MIAT*), gene expression, leukemia

## Abstract

Myocardial Infarction Associated Transcript (*MIAT*) is a nuclear long non-coding RNA (LncRNA) with four different splicing variants. *MIAT* dysregulation is associated with carcinogenesis, mainly acting as an oncogene regulating cellular growth, invasion, and metastasis. The aim of the current study is to investigate the role of *MIAT* in the regulation of T and chronic myeloid leukemic cell survival. To this end, *MIAT* was silenced using *MIAT*-specific siRNAs in leukemic cell lines, and functional assays were performed thereafter. This investigation also aims to investigate the effects of *MIAT* silencing on the expression of core genes involved in cancer. Functional studies and gene expression determination confirm that *MIAT* knockdown not only affects short- and long-term survival and the apoptosis of leukemic cells but also plays a pivotal role in the alteration of key genes involved in cancer, including *c-MYC* and *HIF-1A*. Our observations suggest that *MIAT* could act as an oncogene and it has the potential to be used not only as a reliable biomarker for leukemia, but also be employed for prognostic and therapeutic purposes.

## 1. Introduction

Recent advances in RNA-based approaches and Next Generation Technologies have unveiled the existence of non-coding RNAs (ncRNA), transcripts that lack the ability to code for proteins [1,2]. ncRNAs typically lack an open reading frame (ORF) and are subdivided into housekeeping and regulatory transcripts. Housekeeping ncRNAs are usually 50–500 nucleotides (nt) in length [3], while regulatory ncRNAs are further subdivided in two major classes; small non-coding RNA (sncRNA), classified as 200 nt in length, and long non-coding RNA (lncRNA), which are ≤200 nt in length [4]. LncRNAs share similarities with mRNAs; they are both transcribed by the enzyme RNA Polymerase II (RNA Pol II), have the capability to undergo alternative splicing due to their non-coding exons and undergo post-transcriptional modification such as capping at their 5′ end and polyadenylation at their 3′ end [5,6]. LncRNAs are able to regulate various normal cellular and molecular mechanisms, but they have also been associated with the onset and progression of numerous cancer types [7,8].

LncRNAs are further classified based on their localization, which is usually cytoplasmic or nuclear [9]. A major nuclear lncRNA extensively associated with cancer progression and prognosis is Myocardial Infarction Associated Transcript (*MIAT*), a nuclear retained lncRNA, also known as Gomafu, Retina non-coding (*RNCR2*) or *LINC00066*. *MIAT* was first associated with a higher risk of Myocardial Infarction during a genome-wide association studies [10,11]. *MIAT* is located on chromosome 22q12.1, has a ~10 kb length, and its transcript contains five exons and is polyadenylated [12,13]. The mature *MIAT* transcript is retained in the nucleus and displays a punctuated pattern or spots that do not colocalize with any known nuclear domains [14,15]. 

*MIAT* was found to regulate post-transcriptional processes, acting mainly as a competitive endogenous RNA (ceRNA) [12]. In fact, increasing evidence shows that *MIAT* and mRNAs compete for the binding of various microRNAs (miRNA); generally, *MIAT* interacts with the 3′UTR of a target mRNA, sponging the miRNA of interest. This, in turn, inhibits the translation and generation of end-products, ultimately regulating protein expression [16,17]. Increasing evidence connected the dysregulation of the expression of *MIAT* with carcinogenesis and the progression of various cancer types, highlighting the pivotal oncogenic or the tumor-suppressive role of this lncRNA [13].

Little is known about the regulatory behavior of *MIAT* and its potential role in T and chronic myeloid leukemic cells. Recent evidence has displayed that *MIAT* is upregulated in lymphoid cell lines with mature B cell phenotypes and that *MIAT* created a regulatory mechanism with the protein OCT4 (octamer-binding transcription factor 4), allowing apoptosis and cell death evasion, ultimately promoting cancer progression [18,19]. In addition, further research highlighted the importance of *MIAT* in progression of acute myeloid leukemia (AML) as it was reported to sponge miR-495, affecting further downstream target protein production [20]. Nevertheless, little is known regarding the effects of *MIAT* dysregulation and how the modulation of its expression is potentially relevant to cancer progression and prognosis. To this end, the aim of the current study is to investigate the role of the nuclear lncRNA *MIAT* in regulating the survival of T and chronic myeloid leukemic cells and the expression of different cancer core genes.

## 2. Results

### 2.1. Effects of MIAT Knockdown on the Survival of Leukemic Cells

**Figure 1:**
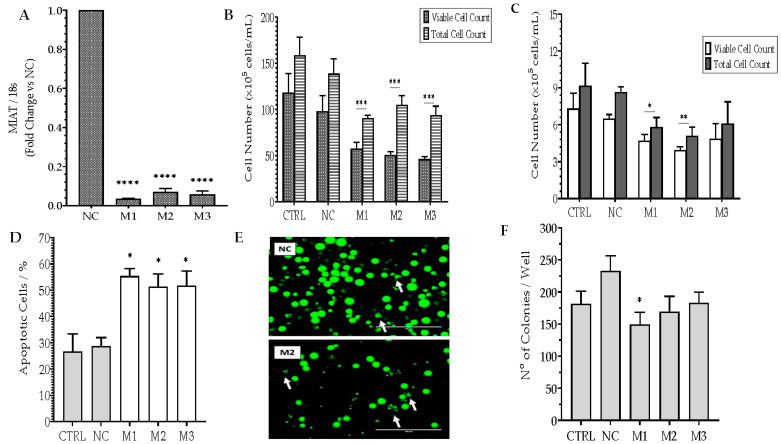
*MIAT*-specific silencing inhibits short- and long-term survival and increases the apoptosis of Jurkat cells. Jurkat cells were transfected with the negative siRNA (NC) or one of the three *MIAT*-specific siRNAs (M1, M2, M3) using Nucleofection, and were assessed 24 h/48 h post-replating. Relative gene expression of *MIAT* was measured by Real-Time PCR 24 h post-transfection and confirmed the silencing of *MIAT* gene for all three siRNAs (**A**). Total and viable cell counts, as determined by vital blue staining, were reduced in Jurkat cells at 48 h post-transfection (**B**). The results were further confirmed utilizing flow cytometry, showing a decrease in total and viable cell count at 48 h (**C**). The rate of apoptosis, determined by acridine orange staining, is increased in *MIAT*-specific siRNAs transfected cells at both 24 h (**D**). Representations of cells undergoing apoptosis are depicted by white arrows (**E**). Long-term survival of Jurkat cells was reduced upon *MIAT*-specific silencing, as demonstrated by clonogenic assay (**F**). Data are represented with bar graphs depicting the means ± SEM from independent experiments. * Indicates a *p*-value < 0.05; ** indicate a *p*-value < 0.01; ***/**** indicate a *p*-value < 0.001, as measured by One-way and Two-Way ANOVA tests and Dunnett’s multiple comparison test (MCT).

To investigate the role of *MIAT* in the regulation of leukemic cell survival, *MIAT* was silenced in Jurkat T cells by nucleofection with negative siRNA (NC) or one of three *MIAT*-specific siRNAs (M1, M2, M3). The effects of *MIAT* silencing upon short- and long-term survival, cell cycle, and apoptosis were assessed. *MIAT* silencing was confirmed prior to further functional studies (Figure 1A). *MIAT* silencing resulted in a significant decrease in both viable and total cells for all three *MIAT*-specific siRNAs (~40% decrease in the number of both total and viable cells in the transfected cells compared to both parental cells and negative control) (Figure 1B). A decrease in short-term survival was further assessed through flow cytometry, which showed a decrease of both viable and total cell numbers in *MIAT*-transfected cells compared to the control with statistically significant results for M1- and M2-specific siRNAs (Figure 1C). Apoptosis rates were assessed using acridine orange staining (Figure 1E). All three *MIAT*-specific siRNA transfected cells showed a significant 2-fold increase in apoptotic cells compared to the control at 24 h (Figure 1D). The long-term survival of Jurkat T cells was measured by anchorage-independent clonogenic assay, and *MIAT* downregulation led to a visible decrease in the colony formation capabilities of transfected cells with statistically significant results for M1-specific siRNA (Figure 1F). To determine whether the decrease in short- and long-term survival of transfected Jurkat T cell was attributed to cell cycle arrest, cell cycle analysis was performed using propidium iodide staining and flow cytometry. The analysis determined that *MIAT* silencing did not have an effect on cell cycle progression (Results not shown). 

**Figure 2:**
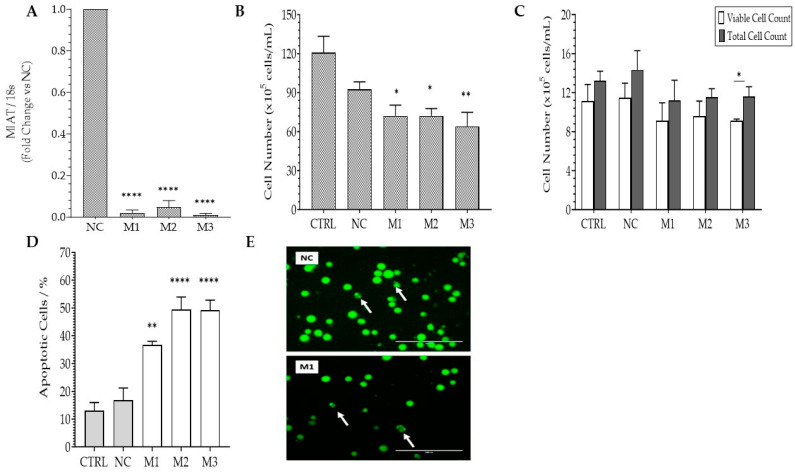
*MIAT*-specific silencing inhibits short-term survival and increases the apoptosis of CEM-C7 cells. CEM-C7 cells were transfected with the negative siRNA (NC) or one of the three *MIAT*-specific siRNAs (M1, M2, M3) using Nucleofection, and were assessed 24 h and 48 h post-replating. Relative gene expression of *MIAT* was measured by Real-Time PCR 24 h post-transfection and confirmed the silencing of *MIAT* for all three siRNAs (**A**). Total and viable cell count, as determined by vital blue staining, was reduced in CEM-C7 cells at 24 h post-transfection (**B**). Further confirmation by flow cytometry showed a decrease in total and viable cell count at 48 h (**C**). The rate of apoptosis, determined by acridine orange staining, is increased in *MIAT*-specific siRNAs transfected cells at 24 h (**D**). Representation of cells undergoing apoptosis are depicted by white arrows (**E**). Data are represented with bar graphs depicting the means ± SEM from independent experiments. * Indicates a *p*-value < 0.05; ** indicates a *p*-value < 0.01; **** indicates a *p*-value < 0.001 as measured by One-way and Two-Way ANOVA tests and Dunnett’s multiple comparison test (MCT).

The results observed in Jurkat T cells were further confirmed with CEM-C7 cells, confirming the growth inhibitory effects of *MIAT* silencing. *MIAT* silencing was confirmed in all *MIAT*-specific siRNAs (Figure 2A) through real-time PCR. Confirmation of *MIAT* silencing was followed by short-term survival assessment (Figure 2B,C), resulting in a significant decrease of viable cell numbers for all *MIAT*-specific siRNAs transfected cells, while no significant changes were observed for the total cell count. siRNA-mediated *MIAT* knockdown in CEM-C7 cells was associated with a 15–40% increase in basal apoptosis (Figure 2D,E) for all *MIAT*-specific siRNAs, confirming the results observed for Jurkat T cells. Cell cycle analysis showed that *MIAT* silencing has no effect on cell cycle progression. This indicates that the increase in the apoptosis rate is responsible for the suppression of growth (Results not shown). 

**Figure 3:**
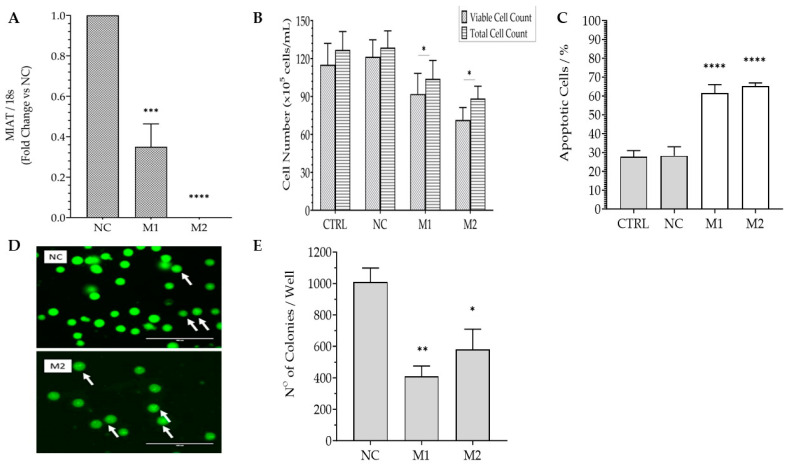
*MIAT*-specific silencing inhibits short- and long-term survival and increases the apoptosis of K562 cells. K562 cells were transfected with the negative siRNA (NC) or one of the two *MIAT*-specific siRNAs (M1, M2) using Nucleofection, and were assessed 24 h and 48 h post-replating. Relative gene expression of *MIAT* was measured by Real-Time PCR 24 h post-transfection and confirmed the silencing of *MIAT* gene for M1 and M2 siRNAs (**A**). Total and viable cell counts, as determined by vital blue staining, were reduced in K562 cells at 48 h post-transfection (**B**). The rate of apoptotic cells, determined by acridine orange staining, is increased in *MIAT*-specific siRNAs transfected cells at 24 h (**C**). Representation of cells undergoing apoptosis is depicted by white arrows (**D**). Long-term survival of K562 cells was reduced upon *MIAT*-specific silencing as demonstrated by clonogenic assay (**E**). Data are represented with bar graphs depicting the means ± SEM from independent experiments. * Indicates a *p*-value < 0.05; ** indicate a *p*-value < 0.01; ***/**** indicate a *p*-value < 0.001 as measured by One-way and Two-Way ANOVA tests and Dunnett’s multiple comparison test (MCT).

To further confirm the findings of both AML cell lines, chronic myelogenous leukemia cell line K562 was also transfected with *MIAT*-specific siRNAs. Of the three *MIAT*-specific siRNAs used, only M1 and M2 led to the downregulation of *MIAT*, as assessed by qRT-PCR in these cells (Figure 3A), and therefore M3 was excluded from further functional analysis. Consistent with previous Jurkat T cells and CEM-C7 results, a decrease in short-term survival of transfected cells was observed in the cells transfected with *MIAT*-specific siRNA compared to the control (Figure 3B). Similarly, flow cytometry assessment revealed 60–80% growth inhibition for cells transfected with *MIAT*-specific siRNAs compared to the negative control (NC) (Figure 3C). Similar to the response in previously assessed AML cell lines, *MIAT* downregulation significantly increased the level of apoptosis by two-fold (Figure 3D,E). In terms of long-term survival, the number of colonies significantly decreased for cells transfected with both *MIAT*-specific siRNAs (Figure 3F) (40% for M1 and 60% for M2). In summary, *MIAT* downregulation decreases the survival of the three leukemic cells lines and triggers a higher rate of apoptosis compared to the controls. 

### 2.2. Effects of MIAT Knockdown on the Dysregulation of Oncogenes and Tumor Suppressor Gene Expression

**Figure 4:**
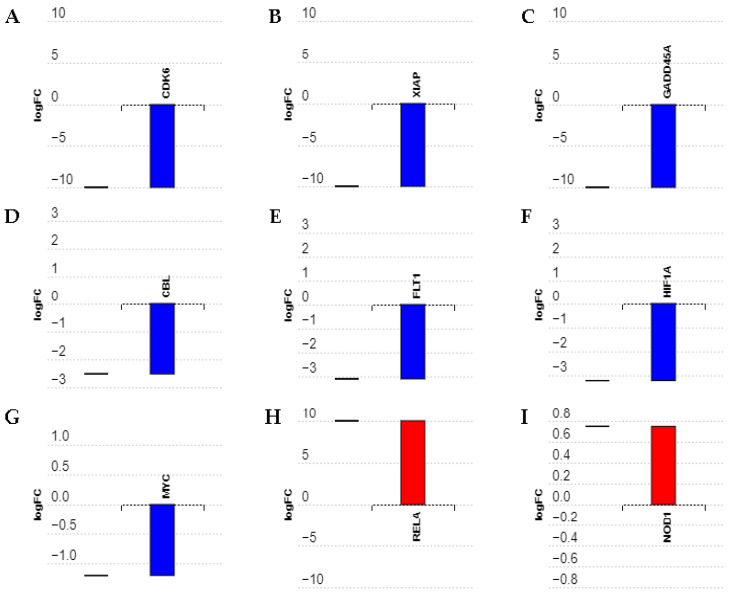
*MIAT* silencing induced dysregulation of several genes in SH-SY5Y neuroblastoma cells. RNA sequencing previously performed showed differentially expressed (**D**,**E**) genes upon *MIAT* silencing [14]. Data are the difference in expression between SH-SY5Y neuroblastoma cells transfected with negative siRNA and cells transfected with *MIAT*-specific siRNA (M2), expressed as a normalized log2 fold change (log2FC). Blue bars represent downregulated genes, red bars represent upregulated genes. (**A**) *CDK6*: Cyclin-dependent kinase 6; (**B**) *XIAP*: X-Linked Inhibitor of Apoptosis; (**C**) *GADD45A*: Growth Arrest and DNA Damage Inducible Alpha; (**D**) *CBL*: Casitas B-lineage Lymphoma; (**E**) *FLT1*: Fms Related Receptor Tyrosine Kinase 1; (**F**) *HIF-1A*: Hypoxia Inducible Factor 1 Alpha Subunit; (**G**) *c-MYC*: MYC Proto-Oncogene; (**H**) *RELA*: *RELA* Proto-Oncogene, NF-KB Subunit; (**I**) *NOD1*: Nucleotide Binding Oligomerization Domain Containing 1.

**Figure 5:**
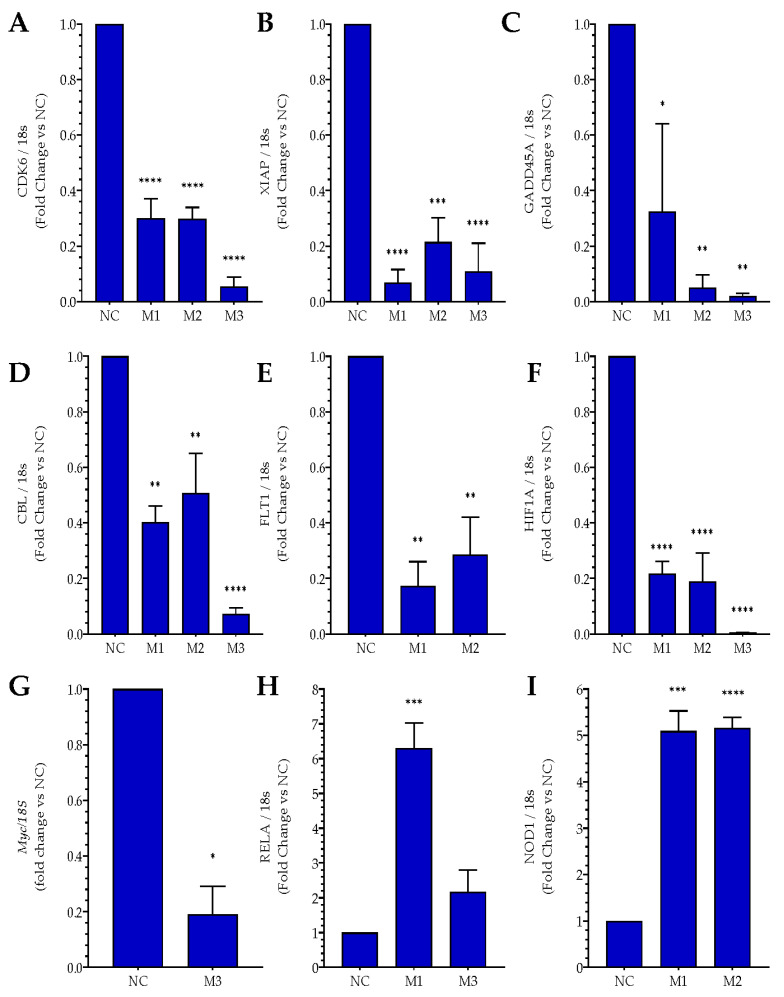
*MIAT* knockdown induces changes in the relative expression of leukemic-related genes. Jurkat T cells were transfected with negative siRNA (NC) or one of the three *MIAT*-specific siRNAs (M1, M2 or M3) using nucleofection. The relative gene expression was measured by Real-Time PCR 24 h post-transfection *CDK*6 (**A**); *XIAP* (**B**); *GADD45*A (**C**); *CBL* (**D**); *FLT*-1 (**E**); *HIF-1A* (**F**); *c-MYC* (**G**); *RELA* (**H**); *NOD1* (**I**). Data are represented with bar graphs depicting the means ± SEM from independent experiments. * Indicates a *p*-value < 0.05; ** indicates a *p*-value < 0.01; ***/**** indicates a *p*-value < 0.001 as measured by One-Way ANOVA tests and Dunnett’s multiple comparison test (MCT). *CDK6*: Cyclin-dependent kinase 6; *XIAP*: X-Linked Inhibitor of Apoptosis; *GADD45A*: Growth Arrest and DNA Damage Inducible Alpha; *CBL*: Casitas B-lineage Lymphoma; *FLT1*: Fms Related Receptor Tyrosine Kinase 1; *HIF-1A*: Hypoxia Inducible Factor 1 Alpha Subunit; *c-MYC*: MYC Proto-Oncogene; *RELA*: RELA Proto-Oncogene, NF-KB Subunit; *NOD1*: Nucleotide Binding Oligomerization Domain Containing 1.

**Figure 6:**
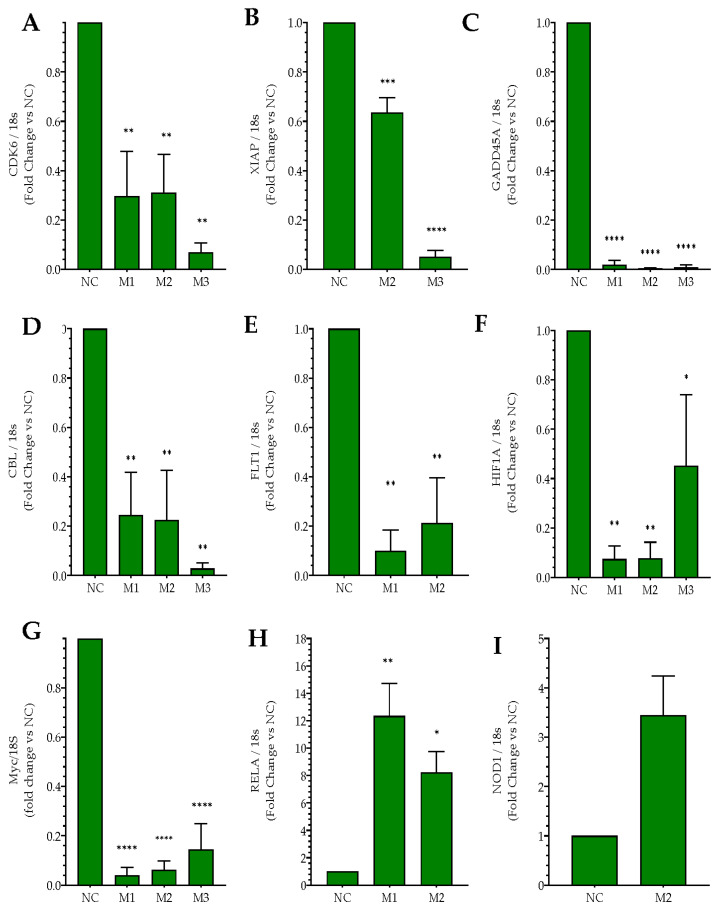
*MIAT* knockdown induces changes in the relative expression of leukemic-related genes. CEM-C7 cells were transfected with negative siRNA (NC) or one of the three *MIAT*-specific siRNAs (M1, M2 or M3) using nucleofection. The relative gene expression was measured by Real-Time PCR 24 h post-transfection *CDK6* (**A**); *XIAP* (**B**); *GADD45A* (**C**); *CBL* (**D**); *FLT-1* (**E**); *HIF-1A* (**F**); *c-MYC* (**G**); RELA (**H**); NOD1 (**I**). Data are represented with bar graphs depicting the means ± SEM from independent experiments. * Indicates a *p*-value < 0.05; ** indicates a *p*-value < 0.01; ***/**** indicates a *p*-value < 0.001 as measured by One-way ANOVA tests and Dunnett’s multiple comparison test (MCT). *CDK6*: Cyclin-dependent kinase 6; *XIAP*: X-Linked Inhibitor of Apoptosis; *GADD45A*: Growth Arrest and DNA Damage Inducible Alpha; *CBL*: Casitas B-lineage Lymphoma; *FLT1*: Fms Related Receptor Tyro-sine Kinase 1; *HIF-1A*: Hypoxia Inducible Factor 1 Alpha Subunit; *c-MYC*: MYC Proto-Oncogene; *RELA*: RELA Proto-Oncogene, NF-KB Subunit; *NOD1*: Nucleotide Binding Oligomerization Domain Containing 1.

RNA sequencing has previously shown that *MIAT*-specific silencing in SH-SY5Y neuroblastoma cells induced dysregulation of an outstanding number of key genes involved in cancer [14]. These genes include major oncogenes and tumor suppressor genes involved in cancer-related pathways as well as cell cycle, apoptosis, and angiogenesis, and in stress signaling. Figure 4 shows the expression of these genes in SH-SY5Y neuroblastoma cells, as determined by sequencing and analysis of the whole transcriptome [14]. Further experiments were carried out to validate RNA sequencing findings (Figure 4A–I). Following siRNA-mediated *MIAT* silencing in both Jurkat and CEM-C7 T leukemic cells, total RNA was collected from transfected cells at 24 h post-replating and gene expression was determined using real-time PCR. The relative gene expressions of Cyclin-dependent kinase 6 (*CDK6*), an important regulatory protein of the cell cycle and also involved in tumor progression; X-Linked Inhibitor of Apoptosis (*XIAP*), a critical regulator of apoptosis; Growth Arrest and DNA Damage Inducible Alpha (*GADD45A*), reported to act as either a tumor promotor or suppressor, and the proto-oncogene Casitas B-lineage Lymphoma (*CBL*) were all significantly lower in *MIAT* siRNAs transfected cells compared to the negative siRNA control. This was confirmed in both Jurkat T-cells (Figure 5A–D) and CEM-C7 (Figure 6A–D). In both Jurkat (Figure 5E) and CEM-C7 (Figure 6E), the growth factor receptor Fms Related Receptor Tyrosine Kinase 1 (*FLT-1*) relative gene expression was also lower than the observed control for cells transfected with M1- and M2-specific siRNAs, however no significant changes were observed for M3 siRNA. Similarly, the expression of Hypoxia Inducible Factor 1 Alpha Subunit (*HIF-1A*), which is associated with tumor metastasis, angiogenesis, and poor prognosis, was significantly lower in the cells transfected with *MIAT* siRNA in both Jurkat (Figure 5F) and CEM-C7 (Figure 6F) compared to the control. These observations further confirmed the RNA sequencing results and the role of *MIAT* in the regulation of gene expression. Interestingly, proto-oncogene *c-MYC* relative expression was also significantly lower in all *MIAT*-specific siRNAs transfected cells in CEM-C7 (Figure 6G), whereas only Jurkat cells transfected with M3 siRNA showed a decrease in *c-MYC* gene expression (Figure 5G). Lastly, RNA sequencing revealed the upregulation of both *RELA* and Nucleotide Binding Oligomerization Domain Containing 1 (*NOD1*), both of which are reported to regulate apoptosis. These results were confirmed in both Jurkat (Figure 5H,I) and CEM-C7 (Figure 6H,I).

## 3. Discussion

The nuclear lncRNA *MIAT* has been shown to be highly dysregulated in a variety of cancer types and also displayed a regulatory role in cell fate decision and survival [13,14]. Nevertheless, despite ongoing efforts to further elucidate the role of MIAT in carcinogenesis and cancer-related processes, little is known about the interconnection between *MIAT* and leukemia. Due to mounting evidence showing the pivotal role of *MIAT* throughout cancer processes, the current study aims to elucidate the functions of lncRNA *MIAT* in T and chronic myeloid leukemic cells. Expression levels of *MIAT* in leukemic samples have been confirmed to be significantly higher compared to normal samples [18,21]. Thus, introducing the hypothesis that downregulation of *MIAT* transcript would influence the proliferation and survival of T and chronic myeloid leukemic cells. To this end, *MIAT* silencing through specific siRNAs indeed caused a significant inhibition of both short- and long-term leukemic cell survival, as well as an increase in apoptotic cell death. These data support a number of independent studies into *MIAT* function. Wang et al. [20] found that silencing *MIAT* reduced the viability of human AML cells and increased apoptosis. Similarly, Sattari et al. [18] showed that *MIAT* knockdown through siRNAs induced an increase in apoptosis in lymphoblastic cell lines and affected caspase 3 and 7 activities. In line with these observations, along with the results obtained by Bountali et al. [14], whereby RNA sequencing revealed that *MIAT* silencing in SH-SY5Y neuroblastoma cells leads to the dysregulation of a diverse number of genes involved in cancer-related processes, the present study confirmed the role of *MIAT* in regulating the expression of core cancer genes, including *CDK6*, *XIAP*, *CBL*, *FLT*, *HIF-1A*, *c-MYC*, *RELA*, and *NOD1.*

Multiple studies demonstrated the importance of Cyclin-dependent kinase 6 (CDK6) for cell cycle progression in cancer cells, placing this gene in the oncogene category [22,23,24]. CDK6 expression showed dysregulation upon *MIAT* knockdown, as shown by Bountali et al. [14]. Our results also show that *MIAT* silencing is associated with downregulation in *CDK6* levels of expression. *CDK6* was shown to be a key regulator of the G1-S phase cell cycle transition and its inhibition induced apoptosis in T-cell leukemia/lymphoma [25]. Furthermore, downregulation or complete loss of CDK6 in AML leukemic cell lines not only decreased the rate of carcinogenesis, but also was found to be a transcriptional target of fusion protein Nucleoporin 98 (NUP98), which is often associated with poor prognosis in leukemic cancer patients [26]. Similarly, decreased levels of *MIAT* led to a decrease in the expression levels of both *XIAP* and *GADD45A*. *XIAP*, a well-known inhibitor of apoptosis [27], was found to be overexpressed in various cancer types, including leukemia [28], allowing cancer cells to escape the apoptotic process. *MIAT* downregulation led to the simultaneous downregulation of *XIAP* gene expression, preventing apoptotic pathway inhibition and, in turn, increasing the rate of death in leukemic cell lines. Similarly, *GADD45A* is highly involved in the regulation of stress-related regulation of cell survival and stressful growth arrest conditions, and expression levels were found to be upregulated in breast cancer [29,30]. Nevertheless, the role of *GADD45A* in leukemogenesis remains unclear as it can act both as an oncogene in response to *c-MYC* or as a tumor suppressor in response to *HRAS* [31]. Thus, the observation of *GADD45A* downregulation upon *MIAT* silencing requires further investigation.

Our results also confirmed the downregulation of Casitas B-lineage Lymphoma (*CBL*) gene expression levels upon *MIAT* silencing. *CBL* is involved in a variety of cancer-related processes, including angiogenesis [32] and tumorigenesis [33], holding the major role of proto-oncogene. In addition, *CBL* is found to play an important role in immunosuppression, regulating the function of T cells, and is also capable of inhibiting T cell activation in the absence of CD28 [34,35,36]. For these reasons, *CBL* has been proposed as a potential biomarker and therapeutic target in immunological diseases. Another gene affected by *MIAT* silencing is Fms Related Receptor Tyrosine Kinase 1, or *FLT-1*. *FLT-1*, which was found to be downregulated upon *MIAT* silencing, is a gene encoding for proteins of the vascular endothelial growth factor receptor (VEGFR) family. The role of *FLT-1* has previously been strongly associated with pre-eclampsia and eclampsia, but recent evidence suggests a role in tumorigenesis and angiogenesis [37]. In fact, *FLT-1* was found to be overly expressed in colorectal cancer tissue samples, and its activation was highly correlated to angiogenesis [38]. The oncogenic role of *FLT-1* was also assessed in leukemic cell lines, whereby its upregulation led to the release of VEGFR factors, enabling the recruitment of angiogenic factors and stimulating the growth of new blood vessels [39,40].

Similar observations were found for *RELA* and *NOD-1*. *MIAT* silencing was associated with an increased expression of *RELA* and *NOD1*. RELA protein, a subunit of NF-κB, plays a regulatory function in immune and inflammatory responses [41,42]. RELA activity has been attributed to the promotion and activation of the NF-κB which holds tumor-promoting roles [43,44,45]. However, other studies reported that overexpression of *RELA* was associated with a reduction in tumorigenicity and activation of apoptosis in the MCF7 ADR human breast cancer cell line [46]. Equally, NOD-1 protein was found to phosphorylate NF-κB by binding to the protein receptor that interacts with serine/threonine kinase 2 (RIPK2), enhancing the proliferative and invasive properties of ovarian cancer cell lines [46]. On the other hand, *NOD-1* expression levels were found to be lower in clear cell renal carcinoma compared to healthy tissue [47], posing the question as to whether this gene acts as a proto-oncogene or has tumor-suppressing capacity [48].

Both *HIF-1A* and *c-MYC* were also confirmed to be downregulated upon *MIAT* silencing in leukemic T cells. The role of *HIF-1A* in cancer is well reported, and *HIF-1A* was found to be upregulated in various types of leukemia [49,50,51,52,53]. The upregulation of this gene was also found to activate the NF-κB pathway, and its downregulation led to a decrease in the proliferation and an increase in the inhibition of apoptosis in AML leukemic cell lines [49]. *c-MYC* has been extensively associated with oncogenic functions, carcinogenesis, angiogenesis, chemoresistance, and many more cancer-promoting roles [54,55]. *c-MYC* has also been found to be a potential therapeutic target in leukemia, along with other cancer types [56,57,58]. Interestingly, a molecular crosstalk between *c-MYC* and *HIF-1A* has been reported in cancer progression [59]. The fact that *MIAT* downregulation affects *HIF-1A* and *c-MYC* expression levels indicates a mechanism yet to be unveiled that has the potential to pose *MIAT* as a therapeutic tool to target the *HIF-1A*-c-*MYC* axis.

Linking the previously described observations, it could be speculated that *MIAT* exerts its effects by acting as a ceRNA and miRNA sponge. Previous studies have shown that *MIAT* acts as a sponge for specific miRNAs, sequestering them from their target genes and leading to an increase in the expression of target genes [12]. In gastric cancer (GC), *MIAT* sponges miR-141, leading to an increased expression of DEAD-box RNA helicase 5 (*DDX5*) and influencing GC cell proliferation and migration [60]. Similarly, in liver cancer, *MIAT* interacts with miR-520d-3p and miR-214, affecting *EPHA*2 and hepatoma-derived growth factor (*HDGF*) expression, respectively [61,62]. It could be assumed that when *MIAT* is silenced, its sponging activity is disrupted, leading to the accumulation of target miRNAs within the cell. As a result, these accumulated miRNAs become available to interact with their target mRNAs, ultimately causing a post-transcriptional downregulation of gene expression. Further investigation into the specific miRNA–mRNA interactions affected by *MIAT* silencing would provide valuable insights into the regulatory role of *MIAT* in gene expression control.

In conclusion, this study has unveiled novel findings regarding the role of *MIAT* in the regulation of cell death and survival of leukemic cells and its impact on gene expression. The considerable influence of *MIAT* on leukemic T and chronic myeloid cells highlights the need for further in-depth investigation into its molecular function and involvement in cancer and other diseases. Additionally, it is crucial to conduct additional research regarding the *MIAT*-*HIF-1A*-*c-MYC* regulatory axis. Notably, the observation that *MIAT* silencing decreases the expression of the oncogene *c-MYC*, which is often deregulated in many cancers and is considered ‘undruggable’, provides an exciting opportunity to explore the development of therapeutic materials targeting *c-MYC*.

## 4. Materials and Methods

### 4.1. Cell Culture

Jurkat cell line (Clone E6-1, (ATCC, Manassas, VA, USA), Cat# TIB-152), apoptosis-sensitive cloned CEM-C7 CKM1 cell line (CCRF-CEM, (ATCC, VA, USA), Cat# CCL-119), and K562 cells (ATCC, VA, USA, Cat# CCL 243) were cultured at 37 °C in a 5% CO_2_ humidified incubator in IMDM medium (GIBCO, Loughborough, UK; #12440053), supplemented with 10% heat inactivated fetal bovine serum (FBS) (Sigma Aldrich, Gillingham, UK; #F9665) and 10 mg/mL gentamicin reagent (GIBCO, UK; #15710-064).

Cells were maintained at a density of 1 × 10^5^–1 × 10^6^ cells/mL. All experiments were carried out using cells in their logarithmic growth phase.

### 4.2. RNA Interference Using siRNAs

The experiments were performed using nucleofection as a method of transfection. The siRNAs used included FlexiTube Negative control siRNA (QIAGEN, Manchester, UK; #1027418), and three different *MIAT*-specific siRNAs, (QIAGEN, UK) M1 siRNA (Cat # SI04287423), M2 siRNA (Cat #SI04314919), and M3 siRNA (Cat # SI04344158), targeting different sites of the fifth exon of the full-length *MIAT* transcript (NR_003491 (10,193 bp)) at a final concentration of 10 µM [14,63,64,65]. Cells were transfected with the Ingenio^®^ kit (Mirus, Geneflow, UK) and the programs T-014 (Jurkat), X-001 (CCRF-CEM) and T-016 (K561-ATCC) were used for the Jurkat, CEM-C7, and K562 cell lines, respectively [63,64]. 5 × 10^6^ cells were transfected and were incubated in complete IMDM medium supplemented with 20% heat inactivated FBS and 10 mg/mL gentamicin reagent for 24 h and re-plated at 4 × 10^6^ for subsequent assessment of long- and short-term cell survival and apoptosis [60,61]. The efficiency of transfection for Jurkat cell line was 64–72% as determined by Cy3 labelling (Invitrogen, Oxford, UK; #1632) [14,63,64].

### 4.3. RNA Extraction

The Direct-zol™ RNA Miniprep kit (Zymo Research, Orange, CA, USA; #R2050) was utilized to extract total RNA from transfected cells, following the manufacturer’s protocol. To evaluate both the quality and quantity of the extracted RNA, spectrophotometric analysis was performed using the NanoDrop™ 1000 (Thermo Fisher Scientific, Oxford, UK). Samples with a ratio between 1.8 and 2 were considered to be of high purity [14,63,65].

### 4.4. Reverse Transcription and Real-Time Polymerase Chain Reaction (RT-qPCR)

Effects of the RNA interference through siRNAs upon gene expression levels were assessed by Real-Time PCR (RT-qPCR). Extracted RNA from transfected cells (as per described in Section 4.3) was reverse-transcribed into cDNA using the Omniscript^®^ RT kit (QIAGEN, Manchester, UK), 10 μM random primers (Thermo Fisher Scientific, Oxford, UK; #SO142), and 10 units/μL of RNaseOUT recombinant ribonuclease inhibitor (Invitrogen; 10777019), following the manufacturer’s instructions [14,61,62]. Real-time PCR was subsequently performed utilizing SensiFast^TM^ Probe Hi-ROX kit (Bioline, TN, USA; #BIO-92020) and TaqMan^®^ Gene Expression Assays (Thermo Fisher, Waltham, MA, USA), (Assay code: *MIAT*, Hs00402814_m1; eukaryotic 18S rRNA, Hs99999901; *CDK6*, Hs01026371_m1; *XIAP*, Hs00745222_s1; *GADD45A*, Hs00169255_m1; *CBL*, Hs01011446_m1; *FLT1*, Hs01052961_m1; *HIF-1A*, Hs00153153_m1; c-MYC, Hs00153408_m1; *RELA*, Hs01042014_m1; *NOD1*, Hs01036720_m1; as described previously [14,62]. The AriaMx (Agilent Technologies, Manchester, UK) was used for the measurement of real-time fluorescence and the AriaMX software was used to perform the data analysis. Expression comparisons were made relative to the negative siRNA (NC) of transfected cells, using the 2^−ΔΔCt^ method [14,63].

### 4.5. Cell Survival and Apoptosis Determination

At 24 h post-transfection, cells were seeded at a concentration of 4 × 10^6^ into a 6 well plate, and cells were thereafter assessed to determine cell viability and apoptosis. Cell viability was determined by counting trypan blue solution 0.4% *w*/*v* (Sigma-Aldrich, UK, # T8154) stained samples using a hemocytometer and light microscopy. Cell viability was also determined using a commercial Muse^®^ Count and Viability Kit (Luminex, TX, USA; #MCH100104) and the Muse^®^ Cell Analyzer (Merck Millipore, Darmstadt, Germany), following manufacturer’s protocol [14,63,64,65].

Apoptosis was assessed by examining nuclear morphology under fluorescence microscopy following staining with acridine orange (25 μg/mL) (Sigma-Aldrich, UK; # 235474). Cells with condensed or fragmented chromatin were considered apoptotic [14,63,64].

### 4.6. Cell Cycle Analysis

Cell cycle analysis was performed using the Muse cell cycle kit (Luminex, Austin, TX, USA #MCH1001060) and flow cytometry, as described previously [14,63,64]. Transfected cells were plated at a density of 4 × 10^6^ cells/well in 6-well plates containing 5 mL of fresh cell culture medium. Cells were collected following 24 h incubation and washed with 500 μL of phosphate-buffered saline (PBS) before being fixed in 1 mL of ice-cold 70% ethanol mixed with 30% PBS. The fixed cells were incubated at −20 °C for 3 h. Subsequently, the cells were re-suspended in 200 μL of Muse™ Cell Cycle Reagent and incubated in the dark for 30 min. The data were then acquired using the Muse Cell analyser.

### 4.7. Long-Term Survival Assessment

Long-term survival was assessed by the cells ability to form colonies on soft agar [63,64]. An equal cell number proportion of cells was diluted in 3 mL of IMDM containing 20% heat-inactivated FBS, 10% cell-specific filtered conditioned medium, and 10% noble agar solution 0.5% *w*/*v* (Difco, Omagh, UK; #214230). The newly formed mix containing cells was placed in 6-well plates. Plates were left at RT °C under sterile conditions, allowing the agar to solidify. Lastly, an overlay consisting of 2 mL IMDM complete medium supplemented with 10% cell-conditioned medium was added to the plates. Plates were cultured at 37 °C in a 5% CO_2_ humidified incubator, and the number of colonies formed were counted after 2–3 weeks.

### 4.8. Statistical Analysis

Statistical analyses were conducted using GraphPad Prism 9 (GraphPad Software, Boston, MA, USA). Data are presented as the mean ± SEM, and the “*n*” represents the number of observations, with each transfected sample obtained from separate experiments. For comparisons, unpaired Student’s *t*-test, One-Way ANOVA, or Two-Way ANOVA with Dunnett’s multiple comparison test (MCT) were applied as appropriate. A significance level of 0.05 was set to determine statistical significance. Differences were considered statistically significant when the *p*-value was <0.05 at a 95% confidence level.

## 5. Conclusions

In summary, the current investigation showed that downregulation of the lncRNA *MIAT* negatively affects the short- and long-term survival of T and chronic myeloid leukemic cells while simultaneously increasing the apoptotic rate. In addition, gene expression analysis showed the expression of a variety of core cancer genes as being affected by the silencing of *MIAT*, highlighting the crucial role of *MIAT* in carcinogenesis and cancer progression. Nevertheless, further investigations are required to elucidate and establish the major pathways affected by *MIAT* and their interactions with components within the pathways highlighted.

## Data Availability

Data will be made available on request.
